# Supplementary material for: The genome sequence of the Oak Beauty, Biston strataria (Hufnagel, 1767)
Source: Wellcome Open Res. 2024 Feb 15;9:35. [Version 1] doi: 10.12688/wellcomeopenres.20835.1 (PMC11294811; doi:10.12688/wellcomeopenres.20835.1)
Supplement: Peer review history

# Reviewer report: Reviewer response for version 1

**DOI:** 10.21956/wellcomeopenres.23054.r78834

**Published:** 2024-08-01

**Authors:** Tyler Scott Alioto

**Affiliations:** 1 Fundacion Centro Nacional de Analisi Genomico, Barcelona, Catalonia, Spain

The genome note submitted by Boyes *et al.* reports the genome sequence of the Oak Beauty, *Biston strataria*. The assembly is chromosome-scale and of high quality, meeting the minimum standards recommended by the Earth Biogenome Project. All protocols are appropriate and well-documented. All data conforms with FAIR principles, with read data and assemblies being available in the ENA. Blobtoolkit figures are interactive. Additional QC data are provided at https://tolqc.cog.sanger.ac.uk/, supplementing the figures published in the data note.

Are sufficient details of methods and materials provided to allow replication by others?

Yes

Is the rationale for creating the dataset(s) clearly described?

Yes

Are the datasets clearly presented in a useable and accessible format?

Yes

Are the protocols appropriate and is the work technically sound?

Yes

Reviewer Expertise:

genomics, genome assembly, gene prediction, annotation

I confirm that I have read this submission and believe that I have an appropriate level of expertise to confirm that it is of an acceptable scientific standard.

# Reviewer report: Reviewer response for version 1

**DOI:** 10.21956/wellcomeopenres.23054.r81629

**Published:** 2024-05-21

**Authors:** Kumar Saurabh Singh

**Affiliations:** 1 Wageningen University & Research, Wageningen, Gelderland, The Netherlands

The authors have assembled the genome of the male Oak Beauty moth, *Biston strataria* (Hufnagel, 1767), utilizing advanced genome sequencing technologies such as Pacific Biosciences single-molecule HiFi sequencing and chromosome conformation Hi-C data. They have deposited the raw datasets in the NCBI and included the accessions for accessing this data. While I found the manuscript comprehensive, I have a few comments on areas that were unclear to me or suggestions for further enhancing the clarity and quality of the manuscript.

 1. The introduction discusses other moth species closely related to *B. strataria*. I believe including a phylogenetic tree illustrating the position of *B. strataria* would greatly enhance the manuscript, effectively conveying the rationale for sequencing this moth species.

 2. I couldn't find details regarding the coverage and quantity of sequencing data generated for the Hi-C and RNAseq libraries. Additionally, annotating the Hi-C contact map with chromosome numbers would be helpful for readers.

 3. Regarding the manual assembly correction, if the Hi-C map was utilized, could you provide a graphical representation indicating where corrections were applied within the contact map? Additionally, it was unclear how correcting a single missing join resulted in a significant reduction of 4.35% in scaffold count. I recommend providing more detailed information on this aspect to help readers understand the connection within the correction process.

 3. In the annotation section, there seems to be no explanation regarding whether the RNAseq data was utilized as external evidence for predicting gene models. I recommend adding those details in that section.

Are sufficient details of methods and materials provided to allow replication by others?

Yes

Is the rationale for creating the dataset(s) clearly described?

Yes

Are the datasets clearly presented in a useable and accessible format?

Yes

Are the protocols appropriate and is the work technically sound?

Yes

Reviewer Expertise:

Bioinformatics, genomics, transcriptomics, metabolomics, data-integration

I confirm that I have read this submission and believe that I have an appropriate level of expertise to confirm that it is of an acceptable scientific standard.
